# N-α-acetyltransferase 10 (NAA10) in development: the role of NAA10

**DOI:** 10.1038/s12276-018-0105-2

**Published:** 2018-07-27

**Authors:** Mi-Ni Lee, Hyae Yon Kweon, Goo Taeg Oh

**Affiliations:** Immune and Vascular Cell Network Research Center, National Creative Initiatives, Department of Life Sciences, Ewha Womans University, Seoul, Republic of Korea

**Keywords:** Protein folding, Protein folding

## Abstract

N-α-acetyltransferase 10 (*NAA10*) is a subunit of N^α^-terminal protein acetyltransferase that plays a role in many biological processes. Among the six N-α-acetyltransferases (NATs) in eukaryotes, the biological significance of the N-terminal acetyl-activity of Naa10 has been the most studied. Recent findings in a few species, including humans, indicate that loss of N-terminal acetylation by NAA10 is associated with developmental defects. However, very little is known about the role of *NAA10*, and more research is required in relation to the developmental process. This review summarizes recent studies to understand the function of *NAA10* in the development of multicellular organisms.

## Developmental disorders: Focus on enzyme activity

Further investigations are needed into the role of a key enzyme in biological development and its encoding gene. The enzyme N-α-acetyltransferase 10 (NAA10), encoded by the *NAA10* gene, plays a role in multiple biological processes. While the function of *NAA10* has been studied in cancer, less is known about the roles of the gene and the enzyme during development, according to a review by Goo Taeg Oh and co-workers at the Ewha Womans University in Seoul, South Korea. Mutations in *NAA10* are found in patients with developmental delay, cardiac problems and skeletal abnormalities, while reduced enzyme activity is associated with developmental defects. Mouse studies suggest a role for *NAA10* in neuronal development, bone formation and healthy sperm generation. The impact of variable *NAA10* expression in different organs at different developmental stages needs clarification.

## Introduction

N-α-acetyltransferase 10 (NAA10), the catalytic subunit of N-acetyltransferase A (NatA), a major N-terminal acetyltransferase complex, catalyzes the alpha (N-terminal) acetylation of nascent peptides as a cotranslational modification and epsilon (internal) acetylation of mature proteins (including itself) as a posttranslational modification^1–4^. *NAA10*, which is conserved from yeast to humans and expressed in most cell types, is an important regulator in diverse biological processes, such as cell growth, differentiation, metastasis, apoptosis, and autophagy^4–10^. In mammalian cells, most studies about the function of *NAA1*0 are mainly focused on its relationship with cancer. Meanwhile, the biological significance of *NAA10* is not as well understood, and further studies are needed in the context of a developing multicellular organism. Recently, *NAA10* has been reported to play a critical role in development and human genetic diseases^11–16^. According to these reports, several human genetic diseases have been shown to be associated with *NAA10* mutations, thus highlighting the importance of NAA10 function during biological development. In this review, we mainly focus on NAA10 function during the development of multicellular organisms.

## Expression of NAA10 during embryonic development

*NAA10* is located on chromosome Xq28 in humans and X A7.3 in mouse, and is encoded by 8 exons^17,18^. Alternative splicing of its mRNA produces several isoforms of *NAA10*. There are three mouse variants (mNaa10^198^, mNaa10^225^, and mNaa10^235^) and two human variants (hNaa10^131^ and hNaa10^235^). The mNaa10^225^, mNaa10^235^, and hNaa10^235^ are the functional isoforms that contain the full N-acetyltransferase domain sequence, and they have been the most extensively studied and characterized among the aforementioned variants^18–20^. Additionally, a homologous gene of *NAA10*, termed *NAA11*, has been identified. The human *NAA11* is located on chromosome 4q21.23, and the sequence of hNAA11 protein is 81% identical to hNAA10, whereas mouse *Naa11* is on chromosome 5E3^21^.

**Fig. 1:**
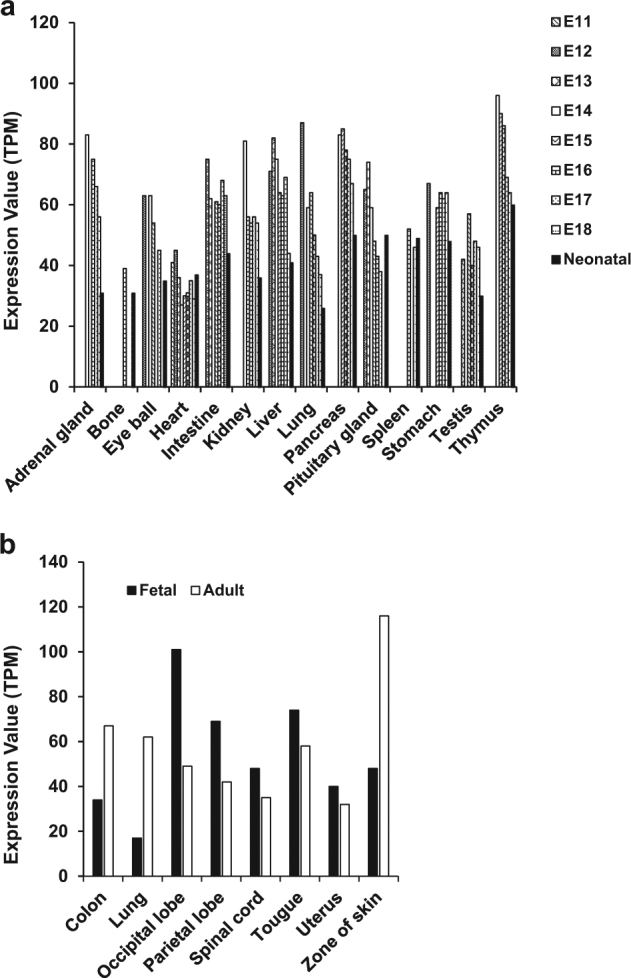
Tissue-specific expression of Naa10 during different developmental stages in C57BL6/J mouse (**a**) and in humans (**b**). Different patterns of expression are observed in each tissue depending on the developmental stage. For the analysis of expression, transcriptome data were extracted from the FANTOM5 Project. TPM transcripts per kilobase million

Previous studies showed the broad and ubiquitous expression of *NAA10* in various cell types, including tumor cell lines and several tissue types, in the developmental stages of embryos and in adults. In general, dynamic changes in the spatiotemporal expression of distinct genes are shown during embryogenesis^22^. The regulation of tissue- and stage-specific expression affects the development of different organs individually^23^. On the basis of this information, it is reasonable to suggest that the potential role of *NAA10* varies depending on transcriptional levels in different tissues and embryonic stages during development. Interestingly, in mouse, according to expression atlas databases, the RNA expression level of *mNaa10* for each organ is higher in the developmental stages of embryo than in the neonate^24,25^. For example, in kidney, liver and lung, the RNA level of *mNaa10*, which is very high in the stage from E12 to E14, decreased to half of this level in the neonate (Fig. 1a). A broad comparison of RNA levels between fetus and adult in humans indicated the same results as those found for mouse^24–27^ (Fig. 1b). However, there are other instances in which an increased or steady expression level is shown in some tissues at the postnatal stage, so we cannot form a definitive conclusion. Whether the changes in tissue- and stage-specific expression have an impact on development needs further investigation.

## N-terminal acetylation dependent functions of NAA10 during embryonic development

Acetylation is one of the protein modifications that diversifies the function of genes in organisms. In particular, N-terminal acetylation occurs in 80~90% of eukaryotic proteins, and the molecular role of N-terminal acetylation affects various protein functions, including protein interaction, localization to the ER and degradation. To date, six distinct forms of NATs (NatA – NatF) have been identified in eukaryotes, and they are classified based on different subunit compositions^28,29^. Among others, NatA undertakes the majority of N-terminal acetylation, acetylating ~40% of the human proteome^10,28–31^. However, the distinct function of NatA has not yet been demonstrated during embryonic development. Nonetheless, abnormal phenotypes were observed in several species due to the absence or reduction of *NAA10*. These data are of great interest for understanding the role of *NAA10* in development.

### Developmental roles in humans

Recently, the importance of NAA10 catalytic activity in human development has emerged through the discovery of various *NAA10* mutations in several pathological conditions (Table 1). Rope et al. reported the first human genetic disorder caused by a Ser37Pro mutation in NAA10 and termed the lethal X-linked Ogden syndrome^14,32^. Since this discovery, more human genetic disorders caused by NAA10 missense mutations, including Tyr43Ser, Arg83Cys, Phe128Leu/Ile, Val107Phe, and Arg116Trp, have been discovered^11,13,15,16^. Among a wide spectrum of malformations in NAA10-related syndromes, there are some common phenotypes with global developmental delay, cardiac anomalies, and intellectual disabilities, and the corresponding *NAA10* mutations triggered a reduction in catalytic activity, suggesting that NATs are essential during early embryogenesis and that they possess important regulatory functions during tissue and organ development^11,33^.

**Table 1:** Summary of phenotypes in human NAA10 mutations

Organism scientific name	Homolog	Type of mutation	Protein effect/molecular mechanism	Phenotype	Ref.
Human Homo sapiens	NAA10	Ser37Pro	Impaired Nt-acetylation in vivo using COFRADIC, reduced catalytic activity for EEEI, DDDI, and SESS, inability to combine with Naa15, reduced degree of Nt-acetylation of THOC7	Perinatal lethal disorder, hypotonia, global developmental delay, cryptorchidism, cardiac arrhythmias, skin laxity, dysmorphic features, hernias, large fontanels	^32^
		c.471+2T→A	STRA6 expression significantly decreased, loss of TSC2 binding and a reduction of TSC2 stability	Eye malformations, mild to severe developmental delay, defects in the skeletal and genitourinary systems, congenital bilateral anophthalmia, postnatal growth failure, skeletal anomalies, hypotonia, moderate-to-severe mental retardation	^12^
		Tyr43Ser	Catalytically impaired in vitro, with approximately an 85% reduction in Nt-catalytic activity for EEEI, DDDI, and SESS	Intellectual disability, facial dysmorphism, scoliosis, long QT	^11^
		Val107Phe/Arg116Trp	Reduction in catalytic activity for the EEEI and SESS (V107P; ~95%, R116W; ~15%)	Severe global developmental delay with postnatal growth, skeletal anomalies, truncal hypotonia with hypertonia of the extremities, minor facial features, behavioral anomalies	^13^
		Arg83Cys	Interfere Ac-CoA binding, 60% reducuction in Nt-catalytic activity	Hypotonia, global developmental delay, dysmorphic features, autism spectrum disorder, epileptic encephalopathy, extrapyramidal signs, hypertension with left ventricular hypertrophy, thin corpus callosum, progressive white matter loss	^16^
		Phe128Leu/Ile	Altered structure and reduced stability, dramatic recuction of Nt-catalytic activity	Moderate to severe intellectually disabled, feeding difficulties, eye anomalies, hypotonia, developmental delay	^15^

Additionally, a splice mutation in the intron 7 splice donor site (c.471+2T → A) of NAA10 was implicated as a cause of Lenz microphthalmia syndrome^12^. Developmental abnormalities, such as microphthalmia or anophthalmia, developmental delay, intellectual disability, skeletal abnormalities and malformations of teeth, fingers, and toes, were observed. Gene expression array studies in patient fibroblasts displayed the dysregulation of several genes involved in embryonic, organ, tissue and skeletal and muscular system development, and above all, these changes were associated with the retinoic acid and Wnt signaling pathways, which are necessary for normal eye development. Furthermore, TSC2 is known to be stabilized by NAA10-dependent N-terminal acetylation. C-terminal truncated mutation of NAA10 demonstrates both loss of NAA10-TSC2 binding and reduced TSC2 protein levels, thereby resulting in perturbed mTOR signaling^12^. Given these findings, the N-terminal acetyltransferase activity of NAA10 plays a critical role in human development.

### Developmental roles in other organisms

In several species, various developmental defects have also been observed due to a deficiency of NAA10 activity (Table 2). *NAA10* was first discovered in yeast, where it was found to be crucial for cell growth and sporulation. The *yNaa10* deleted strain, lacking NatA activity, is viable but exhibits a wide range of defects, including slow growth, de-repression of the silent mating type locus HML (Hidden MAT Left), temperature and salt sensitivity, and failure to enter G0 phase and sporulate^4,7–9^. Recently, the yeast model of Ogden syndrome expressing hNaa10 S37P in *yNaa10* deficient strains has also been shown to have impaired growth and resistance to stress and mating^34,35^. Naa10 is required for *Arabidopsis thaliana* development, and thereby, Naa10 deficient mutations induce growth retardation and are lethal^36^. Additionally, the loss of Naa10 in *Trypanosoma brucei* leads to mortality^37^. In *Caenorhabditis elegans*, daf-31 (the ortholog of *NAA10*) is essential for larval development, metabolism and adult lifespan. Daf-31 mutants fail to properly enter the dauer stage, which is pivotal for *C. elegans* survival during starvation (when nutrients are limited). Furthermore, the mutants display developmental arrest under abundant nutrition and shift their metabolism to fat accumulation^38^. The importance of Naa10 in normal development is also supported by a *Drosophila melanogaster* study in which two hypomorphic mutations of Naa10 led to pleiotropic oogenesis defects, including aberrant mitoses, defects in egg chamber encapsulation and nurse cell chromatin dispersion^39^. Further, *Naa10* deficient mutations have been shown to affect cell survival and proliferation and cause lethality. In a recent *Danio rerio* study, morpholino-mediated knockdown of *Naa10* resulted in increased lethality, growth retardation and abnormal development, such as a bent axis, abnormal eyes, and bent tails, indicating the importance of *Naa10* in early zebrafish development and viability^40^.

**Table 2:** Summary of NAA10 mutations in organisms

Organism scientific name	Homolog	Symbol of mutation	Type of mutation	Protein effect/ molecular mechanism	Phenotype	Ref.
Yeast *Saccharomyces cerevisiae*	ARD1, yNaa10	ard1::HIS3	Inserting a Barn HI fragment containinng the HIS3 gene into the Barn HI site of plasmid YCpE18 that lies within the functional sequence of ARD1	Defect in transcription of a-specific genes, Permit expression of the information resident at *HML*	Reduced viability, sensitive to heat shock and salt, fail to enter stationay phase, lack of glycogen accumulation, sporulation defect, poor mating, fail to undergo meiosis	^8,9^
		nat1-5::LEU; ard1	Mating nat1 and ard1 single mutant for nat1 ard1 double mutant	Impaired N-terminal activity (predicted); single mutants of nat1 (Naa15) and ard1 (Naa10) displayed identical phenotypes, no additional phenotypes are found in double mutant	Inable to sporulate, slow growth, reduced mating, inhibit sporulation, impaired resistance to heat shock, fail to G1 arrest, partial depression of HML, fail to accumulate storage	^4,7^
		y[hNatA S37P]	A strain without yNatA and expression human NatA with a mutated hNaa10 S37P	Lack of proper complex formation with hNaa15 and reduced in vitro catalytic activity, decreased of Nt-acetylome using COFRADIC, increase in the Hsp70 family proteins	Growth defect, sensitive to caffeine and cycloheximide, Impaired resistance to heatshock, decreased mating efficiency	^34,35^
		yS39P	S39P mutation in homologous position to human Naa10 S37		No obious effects	^35^
Plant *Arabidopsis*	At5g13780, AtNAA10	naa10-1	T-DNA insertion-disrupting gene expression	Impaired N-terminal activity; Naa15 mutation also shared same phenotypes	Growth retardation in vegetative stage, lethal, abortion of embryogenesis, drought-adapted root morphology	^36^
		amiNaa10	Depleted-RNA silencing			
Protozoan parasite *Trypanosoma brucei*	TbARD1	Ard1 null mutant	Removal of the ARD1 coding region	Impaired N-terminal activity (predicted)	Impaired growth in bloodstream-form cells, reduced differentiation to insect-stage cells	^37^
Worm *Caenorhabditis elegans*	DAF-31	daf-31(m655)	Remove 151 bp of promoter upstream of the ATG start codon and 242 bp of daf-31 coding region dowstream of the ATG start codon	Regulates the transcriptional activity of DAF-16 (FOXO transcription factor)	Developmental larval arrest, fat accumulation, formation of dauer-like larvae under starvation conditions, decreased lifespan, no SDS-resistant, cannot resume development and reproduction after food re-providing	^38^
		daf-31 RNAi	RNAi knock down; reducing daf-31 mRNA		Decreased lifespan	
		daf-31 OE	Overexpression; full length dar-31 genomic DNA was cloned into pGEM-T		Increase lifespan in daf-2 mutant, enhancing reproduction	
Fruit fly *Drosophila melanogaster*	vnc	vnc^BDk^	Frame shift mutation in acetyltransferase-truncated enzymatic region	Impaired N-terminal activity (predicted)	Pleiotropic oogenesis, aberrant mitosis, egg chamber encapsulation defects, nurse cell chromatin dispersion defects	^39^
		vnc^14^	Copia insertion in the intron			
Zebra fish *Danio rerio*	wufc66b08, zNaa10	naa10MO	Morpholino-based knockdown	Predicted N-terminal activity; zNaa10 has identical substrate specificity to hNaa10 in vitro	Lethality, growth retardation, bent axis and tails, abnormal eyes, less pigmentation	^40^

### Neuronal development

During brain development in mouse, *Naa10* and *Naa15* (the auxiliary subunit of NatA) are highly expressed in regions rich in proliferating and migrating cells, such as the ventricular zone, neocortex, olfactory bulb, and hippocampus^41^. The expression of both genes is down-regulated as neurons differentiate and mitotic and migratory activities subside. Then, once again, their expression increases during postnatal development in the hippocampus and during the neuronal dendritic development of Purkinje cells (PCs) in the cerebellum. This finding indicates that the regulation of expression of both genes is related to neuronal development, especially in the hippocampus and in the PCs of the cerebellum.

The acetylation of α-tubulin has been reported to be involved in regulating microtubule (MT) stability and dynamics, neuron polarization, and neurite branching and promotes vesicular transport on MTs in differentiated neurons^42–46^. A recent study showed that the inhibition of Naa10 or acetyltransferase activity significantly reduced the dendritic extension of cultured neurons^47^. Furthermore, Naa10 and Naa15 proteins co-localized with MTs in dendrites and induced the acetylation of tubulin in the brain fraction. Additionally, the dendritic arborization defect phenotype caused by the over-expression of HDAC6, a major deacetylase of α-tubulin, was rescued by the co-expression of Naa10. Therefore, the authors postulated that Naa10 counteracts HDAC6 by acetylating α-tubulin, thereby promoting MT stability for dendritic development. In spite of this fact, it is not clear that α-tubulin is the only distinct substrate of Naa10 that participates in dendritic development because the tubulin fractions contain both α/β-tubulin. Moreover, it is uncertain whether tubulin is acetylated by the N-terminal activity or lysine activity of Naa10. The lysine 40 residue of either tubulin is de-acetylated by HDAC6^48–50^, so lysine acetylation may be a potential catalytic function of α-tubulin. Characterizing the impact of whichever acetyl activity is involved in tubulin acetylation will help define a specific mechanism for neuronal development.

### Melanogenesis

Naa10 knockdown in Zebra fish 2 days post-fertilization embryos showed less pigmentation, including abnormal development^40^. Pigmentation in organisms is due to the deposition of the pigment melanin, which is produced by specialized cells called melanocytes. In the process that produces melanin, which is called melanogenesis, the most important molecule among melanocyte-stimulating hormones (MSHs) is the proopiomelanocortin (POMC)-derived peptide α-MSH^51,52^. α-MSH is known to be N-terminally acetylated, and its stability and potency in stimulating pigment dispersion are increased by Nt-acetylation^53–56^. So far, N-acetyl transferase enzyme acetylating α-MSH has not been identified yet. NatA is known to acetylate the amino acids starting with Ser, Ala, Thr, Val, Gly, and Cys^28^. Based on this fact, the N-terminus of α-MSH, which starts with serine, demonstrates the possibility that α-MSH could be a potential target of NatA. In the future, more studies are needed to reveal the direct regulation of α-MSH and melanogenesis by Naa10.

### Spermatogenesis

*NAA11* (also known as *Ard1b; ARD2*), a homolog of *NAA10*, is predominantly expressed in mouse^57^ and human^58^ testis. Naa11 can reconstitute functional NAT in the presence of Naa15, where Naa11 is functionally equivalent to Naa10. Interestingly, *NAA10* and *NAA11* display opposite expression patterns during spermatogenesis. In the mouse, the expression of *Naa11* is upregulated during meiosis, whereas *Naa10* expression is downregulated. In contrast, *Naa10* is expressed in pre-meiotic spermatogonia, which do not show *Naa11* expression. Therefore, Pang et al. speculated that compensation for the loss of X-linked *Naa10* occurs by expressing autosomal *Naa11* due to the sex chromosome inactivation during male meiosis^57,59^. In this sense, they also suggested that the NAT activity of Naa10 could play a pivotal role in mitotic spermatogonia and that the NAT activity of Naa11 is crucial for post-meiotic male germ cells^59^. Furthermore, the differential expression of *NAA10* and *NAA11* was shown in the human promyelocytic NB4 cell line upon differentiation^21^; *NAA10* expression decreased with the induction of differentiation in NB4 cells, but the level of *NAA11* remained unchanged, implying that Naa11 has a role in the cellular differentiation process and that Naa10 has a role in the cellular proliferation process. The differential expression pattern of *Naa10*/*Naa11* suggests that *Naa11* is complementary to *Naa10* at least in the mice and that its biological role could be important in spermiogenesis or cellular processes^59^.

## Lysine acetylation dependent functions of NAA10 during embryonic development

Naa10 is known to regulate cellular processes, and its effects are not only catalyzed through its major activity as a NAT but also through the N-ɛ-acetylation of several proteins^2,3,60–73^. The N-ɛ-acetyl-activity of Naa10 requires auto-acetylation^2,67^; this requirement is similar to that of other acetyltransferases, which acetylate themselves for their catalytic and functional activities^74,75^. Recently, gain or loss of function studies in mice have demonstrated that *Naa10* plays an important role in osteoblast differentiation and the early phases of bone formation^3^. Overexpression of Naa10 in mice results in the delayed closure of calvarial fontanels and reduced bone density, osteoblast surfaces and mRNA levels of the osteoblastogenic genes in calvaria. In contrast, *Naa10* deficient mice display calvarial and femoral bone development to a greater extent on postnatal day 3. Mechanically, Naa10 interacts with the RUNT domain of Runt-related transcription factor 2 (Runx2), which is the master regulator of osteoblast development, and acetylates it at K225. The lysine acetylation of Runx2 restricts its transcriptional activity by interfering with CBFβ binding to Runx2. On the other hand, Runx2 stabilizes Naa10 in osteoblasts during bone morphogenic protein 2 (BMP-2)-induced differentiation through its inhibition of IKK-mediated phosphorylation and degradation of Naa10, which in turn inhibits Runx2^3^ (Table 3).

**Table 3:** Summary of NAA10 mutations in mouse

Organism scientific name	Homolog	Symbol of mutation	Type of mutation	Protein effect/ molecular mechanism	Phenotype	Ref.
Mouse *Mus musculus*	Naa10	TgNaa10^235^	Overexpression of Naa10^235^; two different founders #10 and #15 were used	Blocks the Runx2–CBFb interaction by acetylating Runx2 at K225	Delayed calvarial cone development	^3^
		Naa10 KO	Remove Exon1 containing the start codon and Exon2–4 containing N-acetyltransferases, NLS and the Acetyl-coA binding domain	Enhance the the Runx2–CBFb interaction	Facilitating calvarial bone development	^3^
		Naa10^f/Y^; EIIa-Cre	Insertion of loxp into the intron 1 and 6, and the Neo cassette flanked by FRT into the sixth intron before loxp; Cre removes exon 2–6	Disrupts its binding to the imprinted allele at ICRs/DMRs and Dnmt1 recruitment	Partial embryonic lethality, growth retardation, brain disorders, maternal effect lethality, defective genomic imprinting	^88^

In addition to Runx2, the lysine acetyl-activity of Naa10 has been reported to target proteins such as β-catenin (CTNNB1)^60,61^, Phosphoglycerate kinase 1 (PGK1)^72,73^, Hypoxia inducible factor 1α (HIF-1 α)^66–68^, Myosin light-chain kinase (MLCK)^62^, Androgen receptor(AR)^64,65^, enzyme methionine sulfoxide reductase A (MSRA)^63^, SAM domain and HD domain containing protein 1 (SAMHD1)^69^, Heat shock protein 70 (Hsp70)^70^, Aurora kinase A (AuA)^71^, and Naa10 itself^2,67^. The lysine acetyltransferase activity of NAA10 catalyzing these targets has not yet been directly implicated in development, but the targets are essential for developmental signaling pathways, such as Wnt/β-catenin signaling^76–78^, autophagy^79–81^ and the HIF-1 regulatory pathway^82–84^. Accordingly, there is a possibility that NAA10 could influence developmental processes via these signaling pathways. On the other hand, Magin et al. reported data on the lysine acetyl-activity of NAA10, demonstrating that NAA10 does not acetylate the lysine residues of MLCK, MSRA, or RUNX2^85^. For the lysine acetyl-activity of NAA10, earlier reports demonstrated that the auto-acetylation of NAA10 at K136 was a critical step to generate an active form. For example, NAA10 K136R, a mutation in the auto-acetylation site, failed to acetylate β-catenin and did not succeed in the recruitment of β-catenin on the *cyclin D1* promoter^2^. Therefore, the inability of NAA10 to acetylate the lysine residues of MLCK, MSRA and RUNX2 could be due to non-auto acetylated NAA10. There is a possibility that Magin et al. performed their assay with non-auto acetylated NAA10, thus losing lysine acetyl-activity in vitro. Nevertheless, more research is required to understand the role of lysine acetylation by Naa10.

## Acetylation independent functions of NAA10 during embryonic development

Several recent studies have suggested that NAA10 is able to interact directly with other proteins and enhance or inhibit the activity of its partner in an acetylation-independent manner^86^. The signaling pathways of NAA10 and its binding partners have been actively investigated for cell growth and function in cancer research, but reports on acetylation-independent roles of NAA10 during development are very partial as described below.

### Genomic imprinting regulation in embryonic development

Genomic imprinting is an epigenetic process controlled by DNA methylation, and it plays a vital role in normal development. During embryonic development, the establishment of appropriate imprinting is accomplished by the intact regulation of Dnmt3 and Dnmt1 DNA methyltransferases^87^. Lee et al. reported that *Naa10* deficiency mice exhibited developmental defects, including partial embryonic lethality, postnatal growth retardation, brain disorders, and maternal effect lethality, resembling the phenotypes caused by the dysregulation of genomic imprinting^88^ (Table 3). The authors described that despite questions about how Naa10 selectively binds to the imprinted allele, mechanistically, Naa10 binds to the unmethylated GCXGXG in the imprinting control region (ICR)/differentially methylated region (DMR) of the imprinted allele and then recruits Dnmt1 for methylation in the S phase. In addition, by showing the disrupted DNA binding activity of clinical *NAA10* mutations (S37P, V107F, and R116W) to ICR, a potential connection between *NAA10* mutation-associated syndromes and defects in DNA methylation and genomic imprinting was presented. Together, these results suggested that normal DNA methylation and genomic imprinting is regulated by appropriate ICR binding of Naa10 and DNMT1 recruitment during development. Previously, Lee et al. demonstrated that Naa10 acetyl activity does not mediate Naa10-DNMT1 binding and does not stimulate the activity of DNMT1^89^. However, even though Naa10 acetyl activity is dispensable for stimulating DNMT1 activity, we could obtain an insight from their recent study that showed that the N-terminal acetyl-activity of Naa10 is required for binding to ICRs/DMRs. The authors showed that reduced DNA binding to clinically relevant mutations (S37P; defects in Naa15 binding domain for NatA complex, V107F; mutation in the acetyltransferase domain, R116W; putative interference with Ac-CoA binding^15^) are associated with N-terminal acetyltransferase activity, which is not related to the DNA-binding domain at the C terminus of Naa10^9^. Given these results, there is a possibility that the enzyme activity of Naa10 directly or indirectly influences ICR binding. For example, Naa10 may combine with other proteins to acetylate the N-terminal residue, and then, these proteins may interact with ICRs/DMRs together. Interestingly, unlike other reports in other species, including humans, *Naa10* deficient mice exhibited only a few phenotypes, such as embryonic lethality and growth retardation^88^. Moreover, some of the *Naa10* deficient mice developed to term. The authors suggested some reasons for the decreased penetrance of various phenotypes. First, they suggested that this finding was a general effect of imprinting disorders caused by the deficiency of genes that maintain genomic imprinting during global DNA demethylation in zygotes. Second, the remaining DNA methylation occurred at different regions in each individual mouse, which may contribute to stochastic phenotypes. Third, the dysregulation of other imprinted genes, such as *Igf2, Ascl2*, and *Grb10*, and the effects of unidentified non-imprinted genes or protein acetylation by Naa10 deficiency are suggested. In spite of these descriptions, there are still issues to be resolved. It would have been a more complete study if the maternal effects leading to the death of embryos and neonatal mice were explained and if a histological analysis in not only placenta but also whole embryos was performed to determine the precise effects of embryonic lethality. Therefore, more elaborate mechanisms remain to be determined and more extensive mouse studies are needed.

### Vasculogenesis

Xu et al. demonstrate a potential role of Naa10 in vasculogenesis and neurogenesis through the regulation of UNC-5 Homolog B (UNC5B) and Netrin-1 (NTA1)^90^. UNC5B and its ligand NTA1 function as essential genes in morphogenesis of the vascular system or nervous system and the negative regulation of Naa10. Knockdown of Naa10 induced morphological changes (shuttle-shaped membrane protrusion) and increased the ability for tube formation in both H1299 lung cancer cell lines and immortalized mouse endothelial cell lines. Additionally, the expression of UNC5B and NTA1 increased in Naa10 knockdown H1299 cells, and negative regulation was also verified in the mouse caudal half region of E10 embryos (when exuberant vasculature developed at that stage)^90^. However, a direct mechanism explaining how Naa10 regulates NTA1 and its receptor UNC5B has not been determined. Nevertheless, it has been reported that Naa10 physically binds with RelA/p65, an NFκB transcription factor and a negative regulator of NTA1^91,92^. Therefore, the authors speculate that Naa10 physically interacts with p65, thereby negatively regulating NTA1 and its receptor UNC5B.

## Conclusion and perspectives

In this review, we summarized the functions of NAA10 that are enzyme activity-dependent and -independent during the development of several organisms. Most studies have focused on the N-terminal activity of NAA10, whereas some findings have been related to lysine activity and the independent role of NAA10. Our overall understanding of the function of NAA10 in developmental processes suggests the following insights. First, depending on which acetyl-activity is acting and how it affects development, the corresponding physiological responses could be very different. NatA is presumed to act on a large number of substrates involved in a multitude of different cellular processes. As NatA targets many substrates, it could be expected to affect diverse cellular processes and eventually lead to developmental processes in many different organs. On the other hand, since the known targets of lysine acetylation by NAA10 mediate important signaling pathways during development, more intensive and accurate mechanistic studies of these signaling pathways could reveal the role of NAA10 in development. It is also important to understand how the regulation of NAA10 itself is managed during the developmental stage. For example, NAA10 auto-acetylation activates its lysine catalytic activity, and active IKKβ degrades NAA10 by phosphorylation^91^. Furthermore, NAA10 acts as a coactivator of DNMT1 without its enzymatic activity. Likewise, given that NAA10 carries out a wide spectrum of functions, the cellular-, tissue- and developmental stage-specific effects of NAA10 need to be discovered. Second, interestingly, some individuals normally survive and grow with decreased or mutated NAA10, which suggests the possibility of a homologous gene that might compensate for the function of NAA10. This suggestion is supported by the existence of *NAA11*, which has been identified as a homologous gene of *NAA10*, and the reciprocal expression between NAA10 and NAA11 during spermatogenesis. However, we are unable to answer whether NAA11, whose expression appears to be limited to the testis, can cover the entire function of the ubiquitously expressed NAA10 protein. Therefore, an open question about the existence of another homologous gene to *NAA10* in different tissues remains. The goal of future studies will be to elaborate on the cellular/molecular specific activity and distinct pathways of NAA10. Above all, in vivo studies are necessary to analyze the definite biological effects of NAA10.
